# Malaria among febrile patients suspected of Yellow fever during an outbreak in Ghana

**DOI:** 10.21203/rs.3.rs-5684092/v1

**Published:** 2024-12-30

**Authors:** Lidiwan Mensah, Anisa Abdulai, Gloria Amegatcher, Deborah Pratt, Evans Aduhene, Magdalene Sarah Nketia Ofori, Abdul Rahim Mohammed Sabtiu, Patience Adams, Prince Ketorwoley, Christopher Mfum Owusu-Asenso, Nana Aba Setorwu Eyeson, Kwamena William Coleman Sagoe, Joseph Humphrey Kofi Bonney, Yaw Asare Afrane

**Affiliations:** Department of Virology, Noguchi Memorial Institute for Medical Research, University of Ghana; Centre for Vector-Borne Disease Research, Department of Medical Microbiology, University of Ghana Medical School, University of Ghana; Department of Medical Laboratory Sciences, School of Biomedical and Allied Health Sciences, University of Ghana; Department of Virology, Noguchi Memorial Institute for Medical Research, University of Ghana; Centre for Vector-Borne Disease Research, Department of Medical Microbiology, University of Ghana Medical School, University of Ghana; Department of Virology, Noguchi Memorial Institute for Medical Research, University of Ghana; Centre for Vector-Borne Disease Research, Department of Medical Microbiology, University of Ghana Medical School, University of Ghana; Department of Virology, Noguchi Memorial Institute for Medical Research, University of Ghana; Department of Virology, Noguchi Memorial Institute for Medical Research, University of Ghana; Centre for Vector-Borne Disease Research, Department of Medical Microbiology, University of Ghana Medical School, University of Ghana; Centre for Vector-Borne Disease Research, Department of Medical Microbiology, University of Ghana Medical School, University of Ghana; Centre for Vector-Borne Disease Research, Department of Medical Microbiology, University of Ghana Medical School, University of Ghana; Department of Virology, Noguchi Memorial Institute for Medical Research, University of Ghana; Centre for Vector-Borne Disease Research, Department of Medical Microbiology, University of Ghana Medical School, University of Ghana

**Keywords:** Malaria, Yellow fever, Microscopy, PCR, Ghana

## Abstract

**Introduction:**

Between October 2021 and February 2022, there was an outbreak of Yellow fever that spread within several districts in the northern part of Ghana. Febrile illnesses such as Yellow fever are often misdiagnosed as malaria and vice versa, which delays appropriate management and treatment. Hence, the true burden of Yellow fever and malaria are mostly underestimated. This study investigated the epidemiology of malaria in febrile patients suspected of Yellow fever in and around the epicenter of a Yellow fever outbreak in Ghana.

**Methods:**

The study was a cross-sectional study conducted in two outbreak sites (Wenchi and Damongo) and two non-outbreak sites (Kumbungu and Tamale). A total of 498 febrile patients from healthcare facilities were recruited in the rainy and dry seasons. A structured questionnaire was administered to collect patients’ demographic information. Venous blood was collected from consented study participants for malaria parasite detection via microscopy and PCR. Total RNA was extracted from serum samples for the detection of yellow fever virus using Reverse Transcriptase PCR.

**Results:**

None of the patients tested positive for Yellow fever virus. Out of the 498 participants tested for plasmodium parasites, 98 (19.7%) were microscopy positive while 92 (29.7%) were PCR positive. Plasmodium prevalence via microscopy was significantly higher in the dry season, 42 (18.67%) compared to the rainy season, 12 (4.4%) (*P* < 0.001). However, the difference in malaria prevalence via PCR in the rainy and dry seasons was not significant (*P* > 0.05). Kumbungu had the highest parasite prevalence by PCR during the dry (78.38%) and rainy (27.18%) seasons. Higher *Plasmodium falciparum* prevalence was observed using PCR compared to microscopy across all age groups. The age groups with the highest prevalence of *P. falciparum* were under five years (34.69%) and 5 to 9 years (35.56%). The differences in *P. falciparum* prevalence across the age groups were significant (*P* < 0.05).

**Conclusion:**

High malaria prevalence was found in the study sites, affecting preschool and the school-aged children the most. Although Yellow fever was not detected, its overlap with malaria in Ghana, suggests the importance of enhancing surveillance for both diseases to better prevent and control

## Introduction

In Africa, febrile illnesses such as yellow fever are often misdiagnosed as malaria, because they share several symptoms which delays prompt detection and treatment (1). In addition, the true burden of these febrile diseases such as Yellow fever (YF) are understated in Sub- Saharan Africa (2). Symptoms of malaria include fever, chills and headaches and general body aches whilst the intial symptoms of Yellow fever are fever, chills, severe headache, back pain, general body aches, nausea, vomiting, fatigue (feeling tired), and weakness (3). Because most of these symptoms intersect between the two diseases, and the fact that yellow fever outbreaks are sporodic, it is misdiagnosed as malaria (2).

Ghana accounts for 2.2% of global malaria cases and 2% of malaria deaths globally (4). In 2021, an estimated 5.3 million malaria cases with 12,500 deaths were recorded in Ghana according to the World Health Organization (4). Also, Ghana has over the years, experienced several yellow fever outbreaks (5, 6). In a recent outbreak that started from October 2021 to February 2022, a total of 202 suspected cases with 70 confirmed cases and 35 deaths were reported (case-fatality rate 50%). The outbreak was reported in several districts in four regions in Ghana (Savannah, Upper West, Bono and Oti regions) (7). The outbreak started in the Sahel Savannah zone of Ghana (West and North Gonja districts), where malaria prevalence is very high (8). The endemicity of yellow fever and malaria overlap in Ghana (9).

Due to the yellow fever outbreak in Northern Ghana, febrile patients reporting to the hospital may be misdiagnosed as yellow fever and vice versa. Hence, we investigated the epidemiology of malaria and yellow fever in febrile patients suspected of yellow fever in and around the epicenter of a yellow fever outbreak in Ghana.

## Methodology

### Study design and study site description

**Figure 1:**
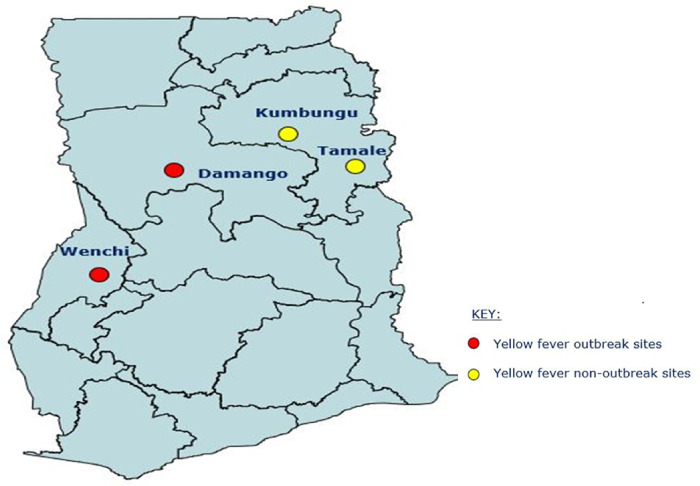
Map of study sites

This study was a cross-sectional study conducted from January 2022 to September 2022 after the outbreak was over. The study was conducted in four districts:, Wenchi (7.7420°N, 2.1008°W) and Damongo (9.3634°N, 1.6761°W) which had outbreak of Yellow fever whilst Kumbungu (9° 22’33.34 “N 0° 42’29.67 “W) and Tamale (9° 33’45.2 “N 1° 01’54.6 “W) were non-outbreak sites close to the outbreaks sites (Figure 1). Damongo, Kumbungu and Tamale are located in the Sahel Savannah zone of Ghana. This zone is characterized by a unimodal rainfall pattern from May to November and a long dry season from December to April. The mean annual temperature ranging from 28 °C to 42 °C. Wenchi is located in the forest zone of Ghana. The forest zone has a tropical rainforest climate, with an annual average temperature of 26.4 °C.

### Study Population

The study population consisted of febrile patients seeking treatment at healthcare facilities located in the selected study sites, namely Tamale Central Hospital, Kumbungu King’s Medical Centre, Wenchi Methodist Hospital, and West Gonja District Hospital. The Tamale Central Hospital is located in the Tamale Metropolis of the Northern Region of Ghana. Kings Medical Centre (KMC) is the only hospital in the Kumbungu district of the Northern region of Ghana. It provides basic health care to the more than 170,000 residents of the Kumbungu district and neighboring districts. Wenchi Methodist Hospital is a 238-bed facility in the Wenchi Municipality. The hospital serves as a referral center for 19 health facilities within the Wenchi Municipality and other districts, Tain and Banda districts. The West Gonja Hospital is located in Damongo, West Gonja District in the Northern Region of Ghana. The hospital is a 100-bed facility and that serves the over 41, 000 residents in the West Gonja District.

### Sample size and sampling technique

A minimum sample size of 358 was calculated based using a prevalence of 37% from Bonney et al. (10) with the formula, $n=Z^{2}P(1-P)/d^{2}$ where, n = required sample size, Z = Confidence level at 95% (standard value of 1.96), P = Estimated prevalence of yellow fever and d= margin of error at 5% (standard value of 0.05). However, 498 febrile participants were recruited for this study. A written consent was obtained from all study participants above 18 years before recruitment into the study. For patients below 18 years, a written consent was obtained from the legal guardians of the minors. In addition, assent was obtained from the minors through signing of an assent form (older minors) or a verbal assent (for younger minors). Recruitment of study participants and blood sample collections were done at two time points in the study sites: the dry season (June 2022) and the rainy season (August 2022 to September 2022).

### Inclusion and exclusion criteria

Febrile patients of any age who visited the healthcare facilities in the selected study sites and provided informed consent or child assent to participate were included in the study. Febrile participants were defined as individuals with a body temperature equal to or higher than 37.5°C. Individuals with a body temperature below 37.5°C were not included in this study. Additionally, febrile patients who were too ill or weak during the data or sample collection period, were excluded from the study.

### Sample and data collection procedure

Venous blood samples of approximately 3 ml were collected from each participant. The blood samples were divided into two parts: one portion was collected into serum separator tubes to obtain serum for the detection of the Yellow fever virus. The remaining portion was used to prepare blood spots and thick and thin blood smears for malaria diagnosis. Dried blood spots was prepared by dropping 50 μl of blood onto strips of Whatman^™^ #3 filter paper. The dried blood spots (DBS) were then be air-dried and placed into zip-lock bags containing silica gel. Also, 100ul of the blood will be used to prepare thick and thin blood smears on microscope slides according to previously described protocols by Dhorda et al. (11).

Blood samples were collected at two-time points: during the rainy and dry seasons. The serum sample were then transported to and analyzed at the Virology Department of the Noguchi Memorial Institute for Medical Research (NMIMR) and the Medical School, all within the University of Ghana, for further analysis. A structured questionnaire was given to participants to collect demographic and other information including, age, gender, address, occupation, vaccination status, travel history, current clinical symptoms, proximity of primates to their residence, and proximity of their residence to vegetation and plantations.

### Molecular Detection of Yellow Fever Virus

Total RNA was extracted and purified from the serum samples of patients using the QIAmp Viral RNA Mini Kit (Qiagen, Germantown, MD, USA) according to the manufacturer’s protocols. A one-step reverse transcriptase real-time PCR reaction was used for the molecular detection of Yellow fever virus using well-described protocols by Domingo *et al*. (12).

### Detection of parasitemia by microscopy

The thick and thin smears were processed and stained with Giemsa (13). Parasites in the thick smears were counted against 200 leukocytes when the slide was positive; otherwise, the whole slide was carefully scanned before being declared negative. Parasite densities were converted to the number of parasites per microliter of blood, assuming a leukocyte count of 8000 cells/μl (14). Parasites in the thick smears were also counted against 200 leukocytes. Stained slides were examined by two independent microscopists to ensure quality.

### Detection of *Plasmodium falciparum* using Nested Polymerase Chain Reaction

DNA was extracted from the dried blood spots on the Whatman filter papers using the Chelex extraction protocol as previously described by Singh *et al*. (15). Extracted DNA was subjected to nested PCR amplifications to detect *Plasmodium infections* according to well-described protocols by Siwal *et al*. (16). Plasmodium genus-specific primers, rPLU5 and rPLU6 were used for the first step of the nested PCR to amplify a 1100 bp PCR product of the 18S rRNA. *P. falciparum* species-specific primers, rFAL1 and rFAL2 in the second nested reaction to amplify a 205bp product indicated a *P*. *falciparum* infection.

### Data analysis

Descriptive analysis were conducted to assess the occurrence, patterns, and socio-demographic characteristics of study participants. Additionally, chi-square tests were carried out at a 95% confidence level to explore the significant differences between socio-demographic factors and malaria infection among the study participants. The parasite prevalence of each population was calculated as the percentage of positive cases over the total examined.

## Results

### Characteristics of the study participants

A total of 498 febrile participants were included in the study during both dry and rainy seasons. The study consisted of a higher proportion of female participants (313, 62.9%) compared to male participants (185, 37.1%). A higher number of participants were recruited in the rainy season (410, 82.3%) compared to the dry season (88, 17.7%). A high proportion of the febrile patients in the study (293, 58.8%) had been vaccinated against yellow fever (Table 1).

### Data represented as frequency (N) and percentage (%)

#### Carriage of Yellow Fever Virus in Febrile Participants

All the 498 febrile participants tested for the presence of yellow fever virus tested negative. The positive control used for this RT-PCR had a CT value of 34.9938 with a red sigmoid curve. Any sample with CT value greater than 40 was considered as negative for yellow fever virus. A total of 293 (58.80%) of the participants had been vaccinated against yellow fever.

#### Malaria infections within the communities

Out of the 498 participants tested for Plasmodium parasites, 98 (19.7%) were microscopy positive while 92 (29.7%) were PCR positive. Females had a higher Plasmodium positivity 83 (16.6%) than men, 46 (9.2%). Plasmodium positivity via microscopy was significantly higher in the dry season, 42 (18.67%) compared to the rainy season, 12 (4.4%) (*χ2* = 25.98, *df* = 1, *P* < 0.001). Similarly, Plasmodium positivity via PCR was higher in the dry season, 65 (28.8%) compared to the rainy season 64 (23.4%), but the difference was not statistically significant (*χ2* = 1.905, *df* = 1, *P* > 0.05).

#### Seasonal variations in Plasmodium falciparum infections across the study sites

**Figure 2:**
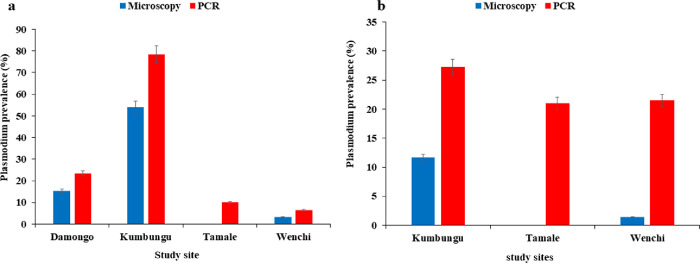
Proportion of participants with malaria parasites; a: percentage of participants positive by microscopy, b: percentage of participants positive by PCR

In the dry season, the highest parasite prevalence was observed in Kumbungu by microscopy 20 (54.05%) and PCR, 29 (78.38%). The lowest parasite prevalence by PCR in the dry season was observed in Wenchi, 2 (6.45%). Similarly, in the rainy season, Kumbungu had the highest parasite prevalence by microscopy, 20 (11.65%) and PCR, 28 (27.18%) respectively (Fig. 2). The differences in parasite prevalence across the study sites were significant (*P* < 0.001).

#### Age variations in Plasmodium falciparum infections

Higher *Plasmodium falciparum* prevalence was observed using PCR compared to microscopy across all age groups. The highest *P. falciparum* prevalence by PCR were observed in the under 5 (35.56%) and 5 to 9 (34.69%) age groups while the other age groups, 10 to 15 and above 15 had parasite prevalence of 33.33% and 21.9% respectively (Fig. 4). The differences in parasite prevalence by PCR across the different age groups of study participants was statistically significant (*χ2* = 10.921 *df* = 4, *P* < 0.05). However, the differences in parasite prevalence by microscopy was not significant (*P* > 0.05).

#### Associations between the carriage of malaria parasites and characteristics of the study participants

Univariate logistic regression analysis of the characteristics of the study participants with the carriage of malaria parasites showed that age and study sites were significantly associated with *P. falciparum* infections (Age, OR = 0.78; Study sites, OR = 0.81, *P* < 0.05) (Table 2). A multivariate mixed-effects logistic regression model showed that age group 5 to 9 years (OR = 1.06) and 10 to 15 (OR = 1.09) had a higher odds of *P. falciparum* infection compared to the under 5 age group. The > 15 years age group was significantly associated with *P. falciparum* infection with a reduced odds (OR = 0.49, *P* < 0.05). For the study sites, Kumbungu had a significantly higher odds of *P. falciparum* infection (OR = 1.98, *P* < 0.05). While a reduced risk of *P. falciparum* infection was observed for Tamale (OR = 1.98) and Wenchi (OR = 1.98) (Table 3).

## Discussion

Febrile illness is a common source of morbidity and mortality in Ghana and Africa (17). However, febrile illnesses such as Yellow fever are often misdiagnosed for malaria, leading to delays detection and affect appropriate management and treatment (2). This study investigated the epidemiology of Yellow fever and malaria in febrile patients in and around the epicenter of a yellow fever outbreak in Ghana. None of the patients tested positive for yellow fever virus, however malaria prevalence was higher through PCR (29.7%) compared to microscopy (19.7%). Malaria prevalence was higher in the dry season compared to the rainy season. The age groups with the highest prevalence of *P. falciparum* the under 5 and 5 to 14 years old.

Although none of the febrile participants tested positive to the yellow fever virus, it is important to strengthen and expand surveillance of yellow fever. It is likely that we did not detect any positive yellow fever cases because of our small sample size. Yellow fever has a short period of viremic phase for the detection of the yellow fever virus by PCR (18). Thus, the use of real-time PCR is difficult to detect the virus after the viremic phase. This highlights the need for more surveillance and control for yellow fever in Ghana.

The overall prevalence of malaria was higher by PCR than microscopy. This is expected because although microscopy is the gold standard for diagnosis of malaria, PCR has been proven to be more specific and sensitive especially for detecting low malaria parasitemia and mixed infections (19). Malaria positivity by PCR was higher in the dry season compared to the rainy season however, the difference was not statistically significant. The highest malaria prevalence during the dry season was observed in Kumbungu. Also, in the rainy season, Kumbungu and Tamale had higher malaria prevalence. Kumbungu is a farming community and has a dam for irrigation. Hence, mosquito-breeding habitats are created when the water held in the dam flows through the conduits to the farmland that it feeds. This may sustain malaria vector populations throughout the year even in the dry season, which explains the high malaria prevalence in the dry season.

Study site, age group and season of sample collection were significantly associated with the carriage of Plasmodium parasites in this study. Children under five and 5 to 9 age groups had the highest malaria infections via PCR compared to other age groups. This is similar to findings from other studies in Ghana (20, 21). This is expected as children under five make up most of the malaria morbidity and mortality in sub-Saharan Africa (22). In Ghana, malaria is responsible for about 20,000 deaths in children annually of which 25% are those aged < 5 years (21). In addition, higher malaria prevalence was observed in the age group 5 to 14 years. This suggests a possible shift of the burden of malaria from children under 5 years to the older age groups (23, 24).

## Conclusion

The study found no evidence of yellow fever virus in febrile patients residing in the epicenter of a yellow fever outbreak in Ghana. However, malaria prevalence was high in the study participants. Interestingly, the areas not affected by the outbreak (Tamale or Kumbungu) had the highest rates of malaria infections. The preschool and the school-age groups had the highest malaria prevalence. These findings calls for more targeted approaches in the control of febrile illnesses such as malaria and yellow fever in Ghana.

## Figures and Tables

**Figure 3:**
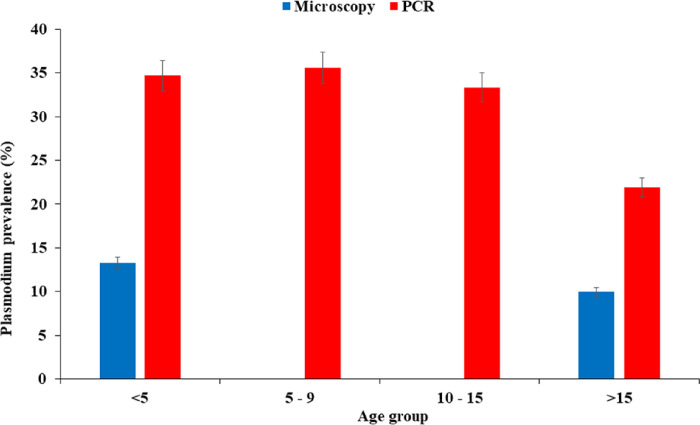
Seasonal prevalence of *P. falciparum* infections across the study sites. **a-b. a:** *P. falciparum* prevalence in the dry season, **b:** *P. falciparum* prevalence in the rainy season. Error bars represent the 95% confidence interval of the mean.

**Table 1:** Characteristics of study participants

Parameter	No. of participants (%)
**Sex**	
Male	185(37.10)
Female	313(62.90)
**Age**	
< 5 years	97(19.48)
5–10 years	46 (9.2)
11–15 years	34 (6.8)
>15 years	321(64.5)
**Study site**	
Tamale	120(24.10)
Kumbungu	140(28.10)
Wenchi	101(20.30)
Damango	137(27.50)
**Season of sample collection**	
Dry	88(17.70)
Rainy	410(82.30)
**Yellow fever Vaccination**	
Yes	293(58.80)
No	205(41.20)

**Table 2:** Univariate association between the characteristics of the study participants and the carriage of *P. falciparum* parasites.

Presence of *P. falciparum* parasitemia	Unadjusted model	
Characteristics	COR [95% CI]	P-value
Age	0.78 (0.67–0.91)	0.002*
Gender	1.09 (0.72–1.65)	0.685
Study site	0.81 (0.68–0.97)	0.023*
Season	0.75 (0.50–1.12)	0.168

* *P* < 0.05

**Table 3:** Multivariate association between the characteristics of thestudy participants and the carriage of *P. falciparum* parasites.

Variables	Multivariate Analysis	
	Adjusted Odds Ratio (95% C.I)	P-value
**Age**		
< 5 years	1.00 (reference)	
5–9 years	1.06 (0.50–2.23)	0.883
10–15 years	1.09 (0.46–2.61)	0.832
>15 years	0.49(0.29–0.85)	0.01*
**Study site**		
Damango	1.00 (reference)	
Kumbungu	1.98 (1.16–3.36)	0.012*
Tamale	0.75 (0.41–1.39)	0.364
Wenchi	0.51(0.26–0.99)	0.049

* *P* < 0.05

## Data Availability

All the data supporting this study are included in the article.

## Abbreviations

DNA: Deoxyribonucleic acid
PCR: polymerase chain reaction
PMI: President Malaria Initiative
WHO: World Health Organization
YF: Yellow Fever
OR: odds ratio
